# Performance Evaluation of Porous Graphene as Filter Media for the Removal of Pharmaceutical/Emerging Contaminants from Water and Wastewater

**DOI:** 10.3390/nano11010079

**Published:** 2021-01-01

**Authors:** Ahmed M. E. Khalil, Fayyaz A. Memon, Tanveer A. Tabish, Ben Fenton, Deborah Salmon, Shaowei Zhang, David Butler

**Affiliations:** 1 College of Engineering, Mathematics and Physical Sciences, University of Exeter, Exeter EX4 4QF, UK; t.a.tabish2@exeter.ac.uk (T.A.T.); bf290@exeter.ac.uk (B.F.); S.Zhang@exeter.ac.uk (S.Z.); D.Butler@exeter.ac.uk (D.B.); 2 Department of Chemical Engineering, Faculty of Engineering, Cairo University, Giza 12613, Egypt; 3 UCL Cancer Institute, University College London, Bloomsbury, London WC1E 6DD, UK; 4 College of Life and Environmental Sciences, University of Exeter, Exeter, Devon EX4 4QD, UK; D.L.Salmon@exeter.ac.uk

**Keywords:** pharmaceutical contaminants, graphene-based materials, porous graphene, adsorption filters, wastewater treatment

## Abstract

Graphene and its counterparts have been widely used for the removal of contaminants from (waste)water but with limited success for the removal of pharmaceutical contaminants. Driven by this need, this study reports, for the first time, the removal of pharmaceuticals from real contaminated water samples using porous graphene (PG) as a filter-based column. This work systematically evaluates the performance of PG as a filter medium for the removal of widely consumed pharmaceutical/emerging contaminants (ECs) such as atenolol, carbamazepine, ciprofloxacin, diclofenac, gemfibrozil and ibuprofen. Several factors were investigated in these column studies, including different reactive layer configurations, bed packing heights (5–45 mm), filter sizes (inner diameter 18–40 mm), adsorbent dosages (100–500 mg-PG) and water bodies (distilled water, greywater, and actual effluent wastewater). Sustainable synthesis of PG was carried out followed by its use as a filter medium for the removal of pharmaceuticals at high concentrations (10.5 ± 0.5 mg/L) and trace concentrations (1 mg/L). These findings revealed that the double-layered PG-sand column outperformed a PG single-layered configuration for the removal of most of the ECs. The removal efficiency of ECs from their solutions was improved by increasing PG dosages and filter bed height and size. Although the treatment of mixed pharmaceutical solutions from different water bodies was affected by the negative interference caused by competing water compounds, the treatment of ECs-contaminated greywater was not severely affected. Our findings suggest that PG, as a highly efficient filter medium, could be used for the removal of emerging pharmaceutical contaminants from water and wastewater.

## 1. Introduction

Pharmaceutical pollution has, in many areas, reached damaging outcomes for ecosystems and living organisms including humans. These drugs have posed various threats to the environment. These contaminants have been defined as “emerging contaminants (ECs)”. Pharmaceutical compounds are commercially produced annually in large quantities (hundreds of tons) for human and animal care [1]; around 200,000 tons of which are only antibiotics [2]. Initially, these pharmaceuticals were designed to possess a biologically active nature, showing persistency and resistance to removal; and therefore, wastewater treatment modalities have very limited success in eliminating them from aqueous environments [3]. Environmental issues exacerbate as a result of the continuous introduction of these pharmaceuticals to water systems via sewage treatment works, livestock waste, and direct application to aquaculture ponds [1,4]. Despite their severe toxicity towards the environment, pharmaceutical manufacturing dispose of high amounts of ECs in the form of industrial wastewater [5,6]. The ecotoxicological effects of these pharmaceutical contaminants (PCs) have been found to be life-threatening [7]. For instance, diclofenac (DCF) at trace concentrations (0.051–0.643 μg/g) resulted in renal failure for vultures in Pakistan [7]. Similar results have been reported for other drugs [4,8].

The presence of pharmaceuticals in drinking water can be a direct result of water-reuse strategies [4]. The conversion of water resources such as wastewater, contaminated freshwater, seawater, greywater, storm water, and brackish water to drinking water as unconventional water supplies has increased recently due to the rising demands on freshwater supplies globally. Nowadays, the gap between wastewater/greywater reuse and drinking water has become narrow due to the recycle/reuse of treated wastewater/greywater as drinking water. The risk posed by these PCs and their transformations into other products is minor at low concentrations (ng/L). However, there is no study dealing with long-term human or environmental health hazards of these PCs contaminated drinking water. Furthermore, it is not acceptable from a general public’s viewpoint to route contaminants of unknown risk to the human body [9]. In response to these concerns, an effective treatment strategy is highly desired to provide the safe and PCs-free drinking water.

Conventionally used wastewater treatments are often limited in the removal of PCs. Advanced treatment technologies are considered as post-conventional treatment processes and tertiary treatment units to activated-sludge-based conventional treatment works to prevent the excessive release of ECs to the environment. Advanced oxidation processes (AOPs), membrane separation, ozonisation, and adsorption are traditionally used treatment technologies that combat ECs in wastewater treatment plants (WWTPs) [10]. Nevertheless, drawbacks are related to the high cost of treatment for most of these technologies and to AOPs that release toxic oxidation intermediates (which require proper control). Among these technologies, adsorption is acclaimed for its low cost, minimal release of toxic by-products, ease of operation, and good reusability of adsorbents [11]. These advantages make adsorption an attractive option for the removal of ECs in tertiary treatment process of WWTPs.

Studies on the impacts of removal technologies in tertiary treatment [12] have revealed that several pharmaceutical compounds are resistant to both granular activated carbon (GAC) adsorption and ozone treatments. The anti-epileptic drug carbamazepine (CBZ) and the lipid regulator drug gemfibrozil (GEM) were not fully eliminated owing to their physicochemical properties, such as high water solubility (for CBZ) and/or poor degradability (of GEM) [13,14]. Concentrations of PCs ranging from 1.7 to 400 ng/L have already been found in drinking water in developed countries, such as the UK, Germany, Canada, Italy and the USA [4]. Besides, pharmaceutical contamination in water is an alarming issue, given that the consumption of PCs in aforementioned countries are much lower than that of highly populated countries such as China, India, Bangladesh, and Pakistan [1].

Nanotechnology has emerged as a promising strategy in solving environmental problems. Graphene-based materials (GBMs), such as graphene oxide and pristine graphene, have a great potential for the removal of pharmaceuticals [11], such as atenolol, ciprofloxacin, carbamazepine, Ibuprofen and many others [11,15,16,17,18,19]. Nanostructured porous graphene (PG) is an ideal water treatment material because of its excellent hydrophobicity, adsorption capacity, recyclability and low toxicity [20]. Besides, its high specific surface area can result in a lower filter volume for water treatment, and its high process efficiency will lead to a lower regeneration frequency [21]. Nevertheless, the application of GBMs for water treatment requires the utilisation of cost-effective, and sustainable synthesis routes. There are many methods to synthesise PG, such as ion bombardment, doping, chemical etching, electron beam irradiation, and chemical vapor deposition [22]. However, there are some drawbacks related to these techniques. For example, they incur high costs with unsatisfactory results. In fact, many researchers have contributed to the development of an alternative method to synthesise PG in recent years. For example, Zhang et al. activated reduced graphene oxide (rGO) with potassium hydroxide to increase its porosity, specific surface area (SSA), graphite layer spacing and adsorption capacity [23]. Unfortunately, the final product turned out to be oxidized porous graphene. In order to solve this problem, our group developed a novel, simple, sustainable, highly biocompatible and cost-effective production route for superhydrophobic PG, based on the heat treatment of rGO [20]. The operation temperature was 190–200 °C, which was lower than that (800 °C) with previously reported synthesis methods. In addition, the surface area of the prepared PG was relatively high (652 m^2^/g).

A few studies explored the potential of GBMs for the removal of ECs (pharmaceuticals in particular), by packed filters in the form of columns. Dong et al. evaluated the efficacy of utilising GO directly as a filter medium for the removal of levofloxacin (LEV), an EC, from its aqueous solution [24]. In fixed-bed columns, GO showed high removal performance for LEV along with lead (Pb) from both single and mixed solutions under different test conditions of GO content and injection flow rate. They concluded that the increase of GO content and decrease of influent flow rate improved the filtration performance and vice versa. Additionally, the competition between LEV and Pb slightly degraded the sorption of GO for LEV. In another report, graphene adsorption reactor (GAR) was coupled with conventional sand filtration to investigate pharmaceutical removal from urban wastewater [21]. Caffeine, carbamazepine, ibuprofen, and diclofenac were successfully eliminated at high concentrations (10 mg/L) of the target pharmaceuticals with removal of more than 95%. Four-month test did not reveal any noticeable typical breakthrough adsorption curves. While comparing GAR to conventional granular activated carbon (GAC) as an adsorbent, graphene filters outperformed GAC filters (96% compared to 62% in the case of GAC). Nevertheless, the application of PG as a tertiary treatment filter for the removal of widely consumed PCs has not been fully explored yet. This exploration is imperative to unlock the current main bottleneck in the GBM limited application in the water treatment sector and influence policy-making as industrial sectors can cost-effectively reduce wastewater emissions by treating effluent wastewaters using GBM technology [25].

In this study, the performance of PG as a filter medium was evaluated for pharmaceuticals removal from water. The removal of widely used pharmaceuticals, namely atenolol (ATL), ciprofloxacin (CIP), carbamazepine (CBZ), Diclofenac (DCF), Gemfibrozil (GEM) and Ibuprofen (IBP) was investigated. Column studies were conducted as an efficient approach towards actual application as a tertiary treatment option [2,3,4]. The study involved experimental tests on the performance of PG as a filter medium for ECs removal from water in column studies under different conditions (reactive layer configuration, size of the column, packing heights, and adsorbent dosage). Moreover, the filtration treatment was investigated for three different types of water bodies (distilled water, synthetic greywater, and actual secondary treatment effluent from wastewater treatment works) spiked with a mixed solution of the six pharmaceuticals. The study setup could assist understanding the effects of water matrix and these contaminants interference with the removal of pharmaceuticals and PG. To the best of our knowledge, the work reported here is the first of its kind to investigate PG material as a nano-adsorbent in column studies for the removal of the six targeted ECs from water.

## 2. Materials and Methods

### 2.1. Materials

#### 2.1.1. Nano-Adsorbent Preparation and Pharmaceuticals 

PG was prepared from graphite (particle size around 20 µm) using the previously reported modified Hummers’ method based on an inexpensive protocol [19,20]. Briefly, graphite flakes were oxidised by concentrated sulphuric acid in the presence of sodium nitrate, potassium permanganate, hydrochloric acid, and hydrogen peroxide, forming graphite oxide which was further exfoliated via sonication for more than 2 h and subsequently reduced with hydrazine. The resultant rGO was exposed to mild thermal treatment at 200 °C in vacuum and left overnight. The final product (PG) had an increased specific surface area and porosity and had proved to be a reliable nano-adsorbent for the removal of conventional contaminants from water [20]. Analytical grade chemicals, including ATL, CBZ, CIP, DCF, GEM and IBP, were purchased from Sigma-Aldrich Co. (Poole, Dorset, UK). They were used to prepare PG precursor solutions and standard stock solutions of pharmaceutical contaminants. LC-MS grade acetonitrile and water were supplied by VWR International Ltd. (Lutterworth, UK), and formic acid (LC-MS grade 99.5+%) was obtained from Fisher Scientific Ltd. (Loughborough, UK). They were used for LC-MS measurements. 

#### 2.1.2. Water Samples

Six pharmaceutical solutions were prepared from each individual pharmaceutical compound in distilled water (DW) with a concentration of 10 mg/L. They were the influent of basic column studies on the effects of different parameters (such as the size of the column, packing heights, and adsorbent dosage) on the PG’s filtration performance. Another solution that contained six ECs spiked into DW medium was prepared for interference studies. The concentration of each EC in these mixtures/multiple contaminants solutions was adjusted to 1 mg/L. Synthetic greywater (SGW) was prepared according to the compositions listed in Table S1 [26]. All the six ECs were spiked into the SGW and the contaminated matrix was the influent to packed column filters for interference studies. 

The properties of the resultant SGW are shown in Table S2, showing its low BOD and COD levels and relatively high content of nutrient contaminants. All the aforementioned chemicals listed in Table S1 were purchased from Sigma-Aldrich Co. (Poole, Dorset, UK) and their grades were of analytical standards. They were utilised to prepare standard stock solutions spiked with multiple ECs in SGW. The concentration of each EC in these mixtures/multiple contaminants solutions was modified to be 1 mg/L. In addition, municipal wastewater (WW) was spiked with multiple ECs. The WW (partially treated) sample was brought from the final settling tank of a secondary wastewater treatment unit (i.e., activated sludge) located in Devon, UK. The water quality of the secondary effluent is shown in Table S3. The extracted WW sample was customised to contain pharmaceuticals as a mixed solution (1 mg/L for each contaminant/EC) and it was used within one week after collection. The sample was refrigerated during that period under 5 °C in a cold store. The six drugs-contaminated water bodies (DW, SGW, WW) were the influents to column filters and were to be treated in interference studies.

### 2.2. Methods

#### 2.2.1. Column Tests

Column studies were carried out to evaluate the performance of porous graphene as filter media for the removal of pharmaceutical/emerging contaminants from water and wastewater. Tests were conducted in duplicates and average results were reported.

#### 2.2.2. Columns Assembly 

**Figure 1:**
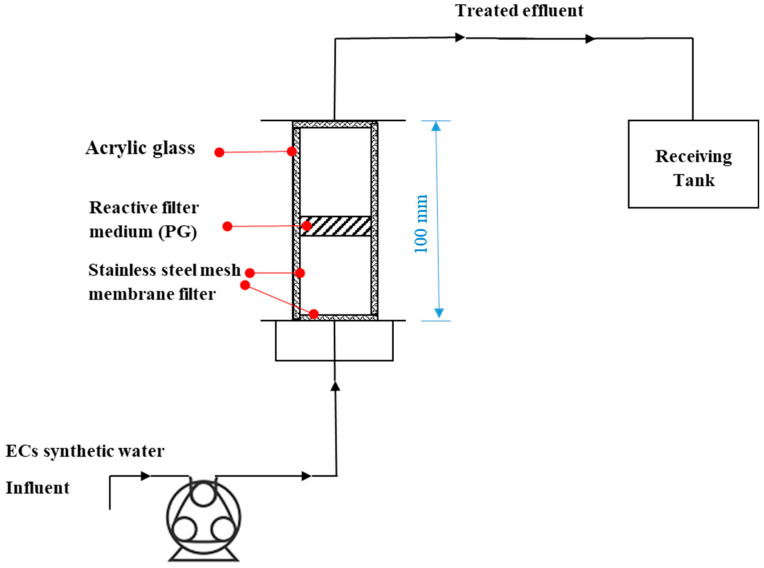
A schematic illustrating of the continuous flow set-up for the column tests.

The columns were made of acrylic glass with sizes of 100 mm in height and 40 mm or 18 mm in inner diameter (ID). They were covered with stainless steel mesh as a filter to prevent particles from escaping out of them. A peristaltic pump (323S/D, Watson Marlow, Cornwall, UK) was used to pump the pharmaceutical solution into each of the columns in an up-flow mode (from bottom to up). The treated effluents were collected in a receiving tank. Figure 1 depicts schematically the set-up for the column study.

#### 2.2.3. Column Sorption Tests

The effect of PG filter on the removal of ATL, CBZ, CIP, DCF, IBP, and GEM from water was quantitatively evaluated by column tests. The interior of each column was filled with a filter bed of pure silica (SiO_2_) sand (50–70 mesh size) purchased from Sigma Aldrich Co. (Poole, Dorset, UK). Stainless steel film (200 mesh) was applied to seal the column and prevent the quartz sand from flowing into the solution after filtering. The top of the column was covered with seal tape to prevent leakage. The first set of column tests was dedicated to studying the effect of column configuration (single or double filter layers of PG). For this first set, three columns (40 mm ID and 100 mm length) were designed and assembled. The first column was completely filled with sand and used as the control column. The second column was filled with sand and an intermediate reactive layer containing 500 mg of PG. The third column was packed with sand for the third of its height; then 250 mg PG was carefully laid flat on the quartz sand; then sand filled again till a height of two-thirds of the column; consequently, another 250 mg PG was filled into the column; and then the rest of column height was packed with sand. The whole column was filled with sand by the wet-packing method. A photograph of the apparatus used for single-contaminant column studies is shown in Figure S1a*,* and an annotated diagram of the column apparatus is shown in Figure S1b.

In the column tests, the first step involved washing of three columns at the same time with deionized water (DIW) at high velocity (30 rpm). After washing, the speed of the peristaltic pump was adjusted to 3 rpm (0.5 mL/min), and the column study was completed in 10 hrs. The samples (3 mL) were taken periodically from the discharge at specific predetermined times for analytical investigations. To achieve a breakthrough condition in the column filters in a reasonable time, the initial concentration of the prepared pharmaceutical (effluent) solutions was high (10.5 ± 0.5 mg/L).

The second set of column tests was carried out using the columns with different internal diameters (ID) (ID 18 mm or ID 40 mm) at the same dosage of PG of 500 mg, and the third set was conducted at different dosages (500 mg PG or 50 mg PG) using 18-mm ID columns or at different dosages (100, 250, 350 or 500 mg) of mixed PG/sand layer (0.5% wt. of PG) using 40-mm ID columns. Investigations on the adsorption performance of PG at different bed heights of fixed PG dosage were carried out in the fourth set. In an 18-mm ID column, the performance of PG was studied using 50 mg PG with two packing heights, 50 mm or 300 mm (PG/SND, 5% wt.). While in the 40-mm ID column, 100 mg PG was used with three packing heights, 18 mm (PG/SND, 5% wt.), 27.5 mm (PG/SND, 0.25% wt.), and 45 mm (PG/SND, 0.1% wt.). Samples were withdrawn at specific periods from the effluent at the top of the column over 600 min. 

A series of interference column studies was also conducted with 100 mg adsorbent dispersed in the filter bed as a slurry. In this case, 100 mg adsorbent PG was dispersed in 25 mL DW, forming a homogeneous slurry. This was then sonicated for 20 min to disintegrate any agglomerations of the adsorbent. Sand was then added to the column in layers and ‘wet packed’ with DW. A layer of slurry was then uniformly dispersed across the sand layer by pipette in 18-mm diameter columns. This process continued until the column was fully packed with sand and the slurry used up. The result was a homogenous distribution of the adsorbent throughout the column. The column was then fully run through for at least two hours with DW to saturate the column. This process was repeated for all the three columns, keeping all variables constant (except adsorbent used). The temperature was kept at room temperature, 22 ± 3 °C, and all solutions were covered to prevent photolysis. The solutions of DW, SGW and WW contained identical EC constituents in the same quantity but differed in that all the six ECs were present in the mixture at a concentration of 1 mg/L each. An appropriate amount of standard pharmaceutical solution (in either DW, SGW, or WW body) was added to its corresponding water body (either DW, SGW, or WW) sample in order to attain the desired initial concentration for each one pharmaceutical compound (1 mg/L). Interference column studies lasted for 300 min and samples were collected regularly at specific times. 

#### 2.2.4. Characterisation Methods 

ATL, CBZ, CIP, DCF, GEM and IBP sample concentrations were determined at 226, 285, 272, 275, 220 and 221 nm, respectively, by using a UV–vis absorption spectrophotometer [21,27,28,29,30,31]. Moreover, lower concentrations of ECs samples (<1 mg/L) were inspected using Liquid Chromatograph Mass Spectrometry (LC-MS). The analysis of these six pharmaceuticals was performed quantitatively using an Agilent 6420B triple quadrupole (QQQ) mass spectrometer hyphenated to a 1200 series Rapid Resolution HPLC system (Agilent Technologies, Palo Alto, Santa Clara, CA, USA). A sample of 5 µL was inserted into a reverse phase analytical column C18 Eclipse Plus (3.5 µm, 2.1 × 150 mm, Agilent Technologies, Palo Alto, Santa Clara, CA, USA). To detect using negative ion mode, two mobile phases were used: mobile phase (A) of LC-MS grade H_2_O (100%) and 0.1% formic acid and mobile phase (B) of absolute LC-MS grade acetonitrile. The following gradient was applied at specific times: 0 min—0% B; 4 min—90% B; 8 min—100% B; 10 min—100% B; 11 min—20% B followed by re-equilibration time of 4 min. The flow rate was adjusted at 0.3 mL/min and the column temperature was kept at 30 °C for the whole period. The electrospray ionisation was carried out under the following QQQ source conditions: gas temperature at 350 °C, 11 L/min drying gas flow rate, nebuliser pressure of 35 psig, and capillary voltage of 4 kV. All ions were scanned in negative ion mode and provided a 30 ms as a dwell time. Prior to the analysis, the fragmentor voltage and collision energies were extensively optimised for each compound (using Agilent Optimiser software) in a series of trials and the optimum values are listed in Table S4, along with the compound retention times (RT) in minutes. Data analysis was performed using Agilent Mass Hunter Quantitative analysis software for QQQ (version B.09.00). Concentrations were calculated using standard calibration curves in the range of 1 mg/L to 0.06 µg/L for each compound. 

Scanning electron microscopy (SEM) and transmission electron microscopy (TEM) images were obtained using SEM-EDS (TESCAN VEGA3 SEM fitted with X-MAXN EDS detector) subject to a high vacuum condition at accelerating voltage 20 kV, and JEOL-2100 transmission electron microscope (TEM) operated at an accelerating voltage of 200 kV, respectively. X-ray diffraction measurements were conducted with a Rigaku diffractometer (Cu Kα radiation, a wavelength of 1.5406 Å; an operating energy of 40 keV; a cathode-current of 40 mA; and a scan rate of 1° min^−1^). The Fourier-transform infrared (FTIR) spectroscopies were performed using a Bruker Optics Tensor-27 FTIR spectrometer. The analysed spectra were acquired between 500 and 2000 cm^−1^ at a 4-cm^−1^ resolution using 25 co-added scans. Raman spectra were measured using Renishaw RM-1000 spectrometer at wavelength of 532 nm excitation operated at power of 6 mW.

## 3. Results and Discussion

### 3.1. Material Properties

**Figure 2:**
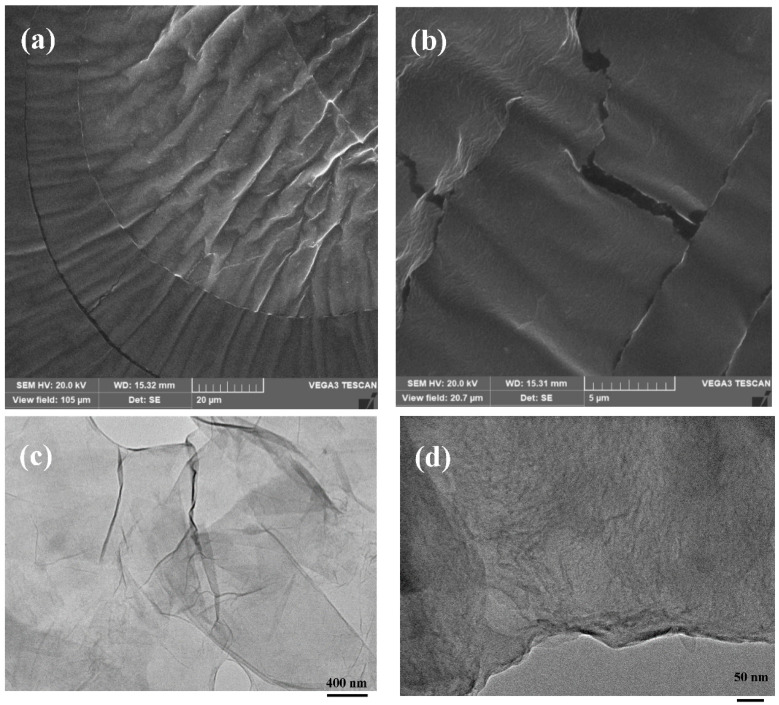
SEM images of as-prepared PG (**a**,**b**) at a magnification of (**a**) 20 and (**b**) 5 µm and High-resolution TEM imaging (**c**,**d**) of PG product at a resolving power of (**c**) 400 nm and (**d**) 50 nm.

High magnification SEM observation of the resulting as-prepared PG powder (Figure 2a,b) reveals the high transparency of graphene sheets. The folding at the sheet’s edges indicates a minute thickness. Corrugated sheets of graphene with wrinkles were observed at regular intervals across the image (Figure 2a). The expected high SSA of PG had a direct link to the appeared morphology in Figure 2. It is worth mentioning the results of surface area characterisation showed a high SSA of 670 m^2^/g (divided into micropore area 312 m^2^/g and external surface area 358 m^2^/g), nano-channels of mean pore size 4 nm and total pore volume of 0.475 cc/g. The nanostructured porous and high surface area property facilitated the aggregation of graphene sheets, which led to the formation of stacked graphitic structures. A magnified scene of such agglomerate is presented in the nanometer domain in the high magnification TEM images of PG nanosheets (Figure 2c,d), showing the features of as-prepared PG nanosheets. From Figure 2c, it is observed that PG nanosheets were entangled with one another, forming a large, transparent silk-like spreadsheet over the carbon-coated copper grid specimen. Corrugations and wrinkles arose from exterior forces acting on a planar graphene sheet. These might be uniaxial or multidirectional, each resulting in different morphological features. From a thermodynamic viewpoint, PG sheets as 2D-membrane structures bend to gain stability [32]. As a result of bending and scrolling of PG sheets, nano-cavities appeared in the PG structure. A closer look at the basal planes of PG nanosheets is shown in Figure 2d where the nano-channels on the plane of stacked graphene nanosheets are visible.

**Figure 3:**
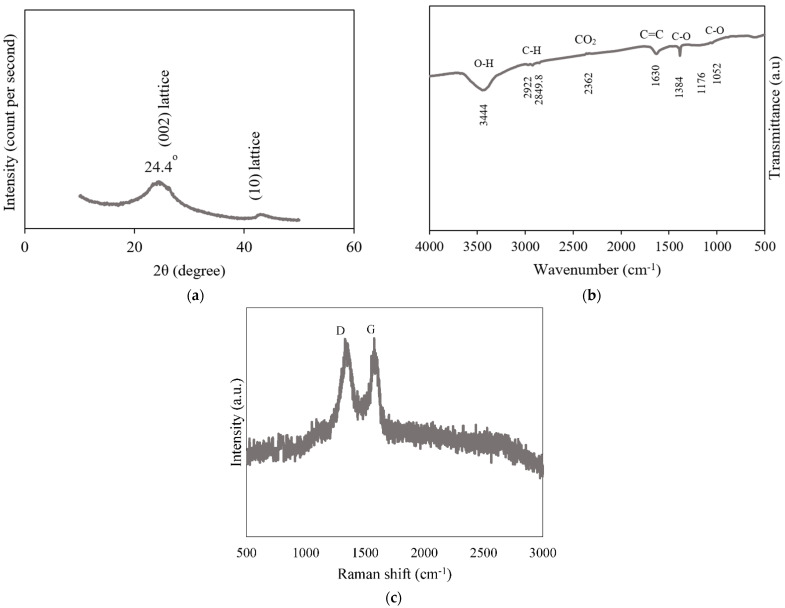
XRD (**a**), FTIR (**b**), and Raman (**c**) patterns recorded for as-prepared PG material.

To characterise the PG material, XRD, FTIR, and Raman spectroscopy were conducted. The XRD spectrum recorded in the range from 10 to 50° (2θ) (Figure 3a) displays a diffraction peak at 2θ = 24.4°. The (0 0 2) plane peak indicates the distance between the graphene layers. The graphene layer distance of (0 0 2) reflection was calculated according to the Bragg’s equation [33] to be 0.37 nm. Given that the peak Full Width at Half Maximum (FWHM) is 7.021 and 2θ is 24.4°, the Scherrer formula with Warren constant of 0.94 was applied to evaluate the average crystallite height of PG stacking layers (1.21 nm). The average diameter of stacking PG layers was estimated by applying the Scherrer equation with a constant of 1.84 to two dimensional (10) lattice reflection. The results indicated that PG was composed of three to four layers in a stacking nanostructure of a mean crystallite diameter by height of around 12.3 nm × 1.21 nm and distance between graphene layers of about 0.37 nm.

The functional groups of graphene were investigated by analysing its FT-IR spectrum [34] (Figure 3b). The peaks at 1384 and 1052 cm^−1^ indicated the existence of C–O stretching vibration. The band at 1630 cm^−1^ was associated with the stretching vibration of C=C groups. The strong peak at 3444 cm^−1^ reflected the stretching vibration of OH groups. The band at 2632 cm^−1^ indicated the existence of asymmetric and symmetric stretching vibration of C=O groups. The band at around 1110 cm^−1^, which indicated the presence of C–N groups from hydrazine hydrate, was not detected, indicating no hydrazine (reducing agent) traces in the produced PG [35]. The bands at 2922 and 2849.8 cm^−1^ were attributed to the C–H bending vibrations. 

The presence of some peaks corresponding to oxygen-containing functional groups in the FT-IR spectrum (Figure 3b) could be ascribed to the nature of the graphene synthesis process. The applied chemical reaction synthesis route does not guarantee the total elimination of oxygen-containing functional groups. PG has an average composition of carbon element exceeding 91% as previously characterised and mentioned in our reports [19,20]. These results are in line with several reports in the literature for the synthesis of PGs [36,37,38]. 

To determine the order and disorder in the crystalline structure in graphene nanosheets, the Raman spectrum of as-prepared PG nanosheets is given in Figure 3c. D and G peaks were visible at around 1350 and 1580, respectively. Unlike graphite materials, a strong D peak is exhibited by graphene defected crystals. For perfect crystalline graphite materials, the D peak is barely visible [39]. The G peak, similar in intensity to the D peak, appeared as an indication of the bond-stretching motion of C sp^2^ atom pairs. The shown spectrum and D/G intensity ratio revealed a graphene nanosheet with partially disordered crystal structure along with ordered in-plane C sp^2^ domain. 

### 3.2. ECs Removal in PG-Sand Columns

The following column studies scrutinised the efficacy of PG material as a filter medium to remove six drugs/ECs, namely ATL, CBZ, CIP, DCF, GEM, and IBP, from water. PG has a proven affinity towards several pharmaceutical contaminants [11,19]. The modelling of adsorption data of PG for the aforementioned pharmaceutical contaminants was previously reported by our group in which the adsorption mechanism was explored in batch tests via thermodynamic studies, adsorption kinetics, and equilibrium isotherm modelling [19]. Kinetic studies showed that the adsorption was guided by the pseudo-second order model, and the majority of adsorption processes by PG for ECs followed the Toth and Sips adsorption isotherm models as a result of hydrophobic interactions and heterogeneous adsorption.

#### 3.2.1. Single and Double-Layered Configurations

**Figure 4:**
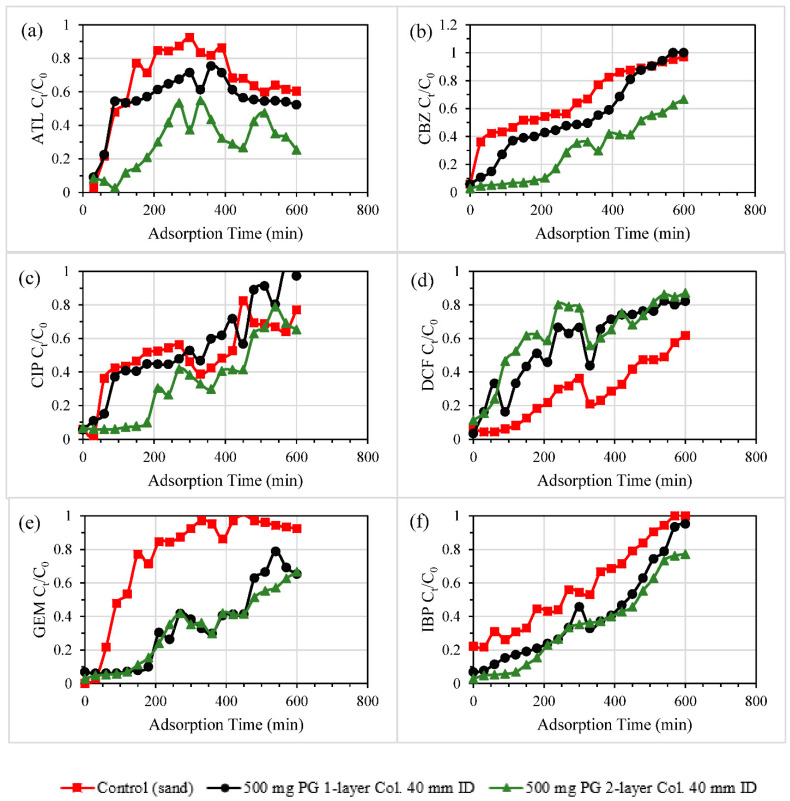
Transport and decontamination of (**a**) ATL, (**b**) CBZ, (**c**) CIP, (**d**) DCF, (**e**) GEM, and (**f**) IBP from EC-contaminated DW (conc. 10.5 mg/L) at flow rates of 0.5 mL/min through sand columns packed with PG in two different configurations (one or two PG layers) compared to a control sand column.

Presented in Figure 4 are results from the basic column studies using different configurations of PG (one or two layers) at a fixed dosage of 500 mg, filtering ECs-contaminated DW of a concentration of 10.5 mg/L. ATL removal was significantly enhanced using a double-layered configuration in a PG-sand column, as demonstrated in Figure 4a. Two layers of PG outperformed a single layer of PG in terms of ATL removal performance. This was similarly the case with CBZ removal (Figure 4b). Even the performance of PG-sand column of two PG layers in GEM and IBP removal was slightly better than that of a single PG layer as depicted in Figure 4e,f. However, breakthrough curves of the three columns were close to one another for CIP removal (Figure 4c). Figure 4d depicts the decontamination of DCF by PG layers which produced different results. DCF removal was improved in the PG-free sand columns. Batch experiments were conducted to explain the affinity of CIP and DCF towards sand in which 20 mL solution of 12 mg/L as a concentration of ATL, CIP or DCF was mixed with specific dosages of sand (0.5, 1, 2, 3 g) and shaken over 24 h. The results of batch tests (Figure S2) revealed the highest affinity and adsorption capacity was for DCF and then CIP, while ATL showed no attraction towards pure silica sand. These results elucidated the outperformance of sand columns in DCF removal column studies (Figure 4d), the close performance of the control sand columns in the case of CIP removal to the others (Figure 4c), and the degraded ATL removal performance by the pure sand filter in Figure 4a. Even the filtration of ATL-contaminated DW by control sand filter (Figure 4a) seemed to be enhanced by the remaining washing water inside the filter, allowing significantly low effluent concentrations of ATL.

Overall, the performance of the three investigated columns for the adsorption of five ECs can be ranked in the following order: double layer PG-sand column > single layer PG-sand column > control sand column. These results could be explained as the adsorption occurred during a longer packing height, and that distance resulted in higher removal efficiency and more EC uptake. Similar multilayer outperformance could be witnessed in previous reports, utilising filters packed with nanomaterials such as nanoscale zero-valent iron treating nutrient (nitrate and phosphate) contamination [40,41].

#### 3.2.2. Size of Columns

**Figure 5:**
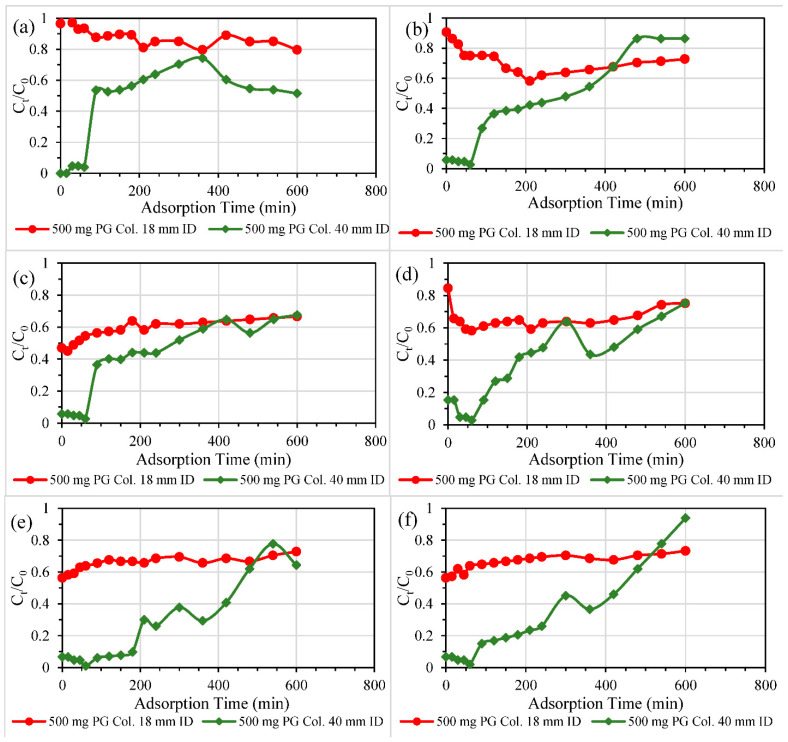
(**a**) ATL, (**b**) CBZ, (**c**) CIP, (**d**) DCF, (**e**) GEM, and (**f**) IBP adsorption onto PG filter medium from contaminated DW (of 10.5 mg/L as EC concentration) using the same dosage (500 mg) in different column sizes.

Column studies were carried out using different column sizes (ID 18 mm or ID 40 mm) at the same dosage of PG of 500 mg, decontaminating DW spiked with specific EC at 10.5 mg/L. Figure 5 illustrates ECs removal performance of these two fixed-bed adsorbers, revealing that the adsorption performance of PG packing was improved in a larger cross-sectional-area column. 

Filters of higher cross-sectional areas (and consequently larger volumes) produced clearer adsorption breakthrough curves and the breakthrough time (around 60 min) was easy to determine from the graphs (Figure 5) in contrast with the results obtained from smaller fixed bed filters. This broad difference in filtration performance can be attributed to the variation in contact time between different size adsorption columns. The contact time with the reactive material (PG) was approximately the same (11.5 min) in both filters. However, the distribution of ECs influent to the PG reactive layer could be more efficient in the case of 40-mm fixed bed filter, while the longer bed length with less homogeneous distribution in 18-mm filter led to degraded performance for the same PG loading. Besides, the amount of washing water, which was left in the columns after the initial washing stage, was larger in 40-mm filters. The remaining water contributed to the dilution of effluent concentrations from large filters. Moreover, the amount of sand used in large filters was much higher than that of small filters, resulting in further improvement in the breakthrough profiles of 40-mm ID adsorber. It is inferred from the treatment profile of small size columns that a hydraulic retention time of around 11 min was not sufficient to reduce the early concentrations of first collected samples to a reasonably reduced concentration. For instance, 80% of the influent concentration still remained in ATL effluent, which showed poor and non-reliable performance. Similar cases were witnessed in the other ECs decontamination profiles. 

#### 3.2.3. Dosage of PG

**Figure 6:**
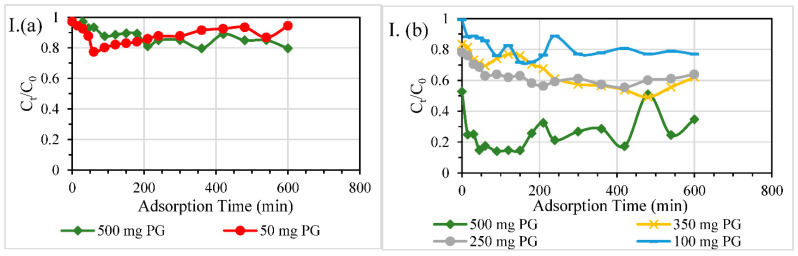

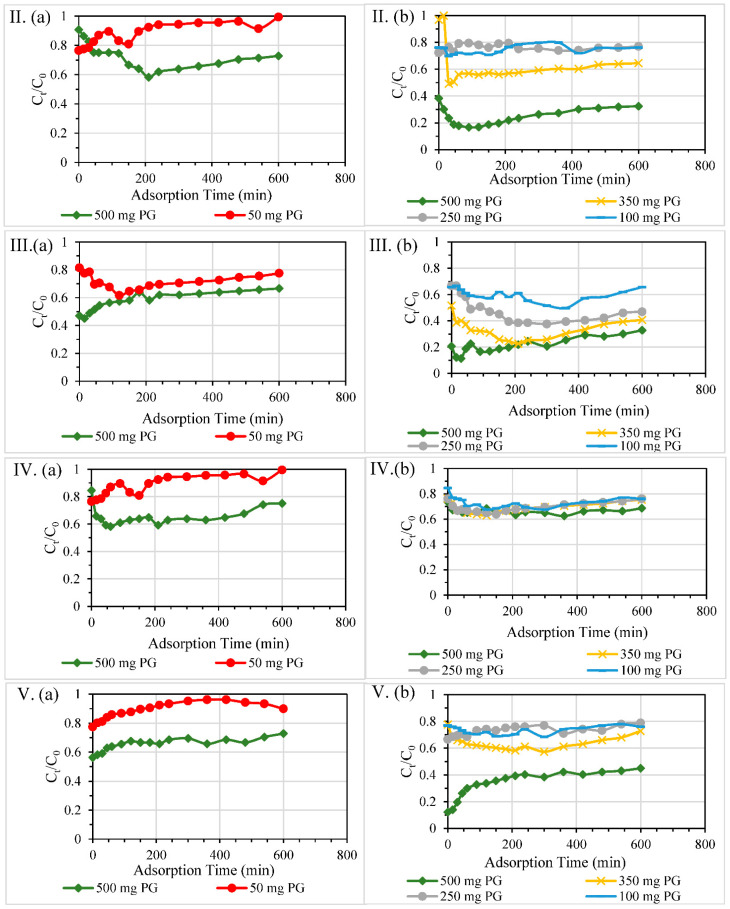

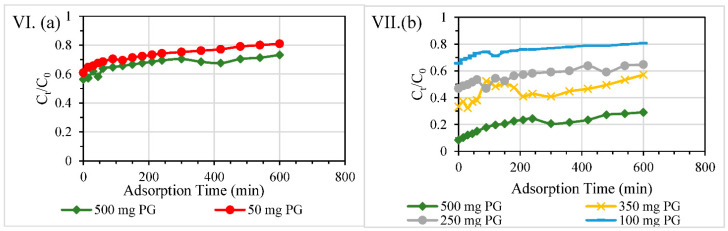
(**I**) ATL, (**II**) CBZ, (**III**) CIP, (**IV**) DCF, (**V**) GEM, (**VI**) IBP adsorption onto PG/SND (PG at 0.5% wt.) filter medium in different column sizes (**a**) 18 mm ID (**b**) 40 mm ID. Experimental conditions: ECs-contaminated DW influent adjusted at 10.5 mg/L of EC; room temperature at 22 ± 3 °C.

Tests were conducted at different dosages (500 mg PG or 50 mg PG) using 18 mm ID columns or at different dosages (100, 250, 350 or 500 mg PG) of mixed PG/sand layer (0.5% wt.) using 40 mm ID columns. The influents to these column filters were contaminated DW prepared by adding specific EC (10.5 mg/L) into DW. The results are shown in Figure 6I–VI. ATL was barely removed using 50 mg-PG in an 18-mm filter (Figure 6I(a)). The increase of fixed bed dose to 10 times slightly decreased the treated effluent concentration and improved maximum removal efficiency to 20%. The degree of improvement was higher in the case of CBZ (Figure 6II(a)), DCF (Figure 6IV(a)), and GEM (Figure 6V(a)). Using another filter size (40 mm ID), a noticeable variation in effluent concentration was observed at different dosages of PG/sand. A reasonable trend in the results was demonstrated as 500 mg PG mixed with sand bed maintained output treated ATL concentrations at about 20% of the initial effluent ATL concentration. The initial drop in ATL concentrations of the effluents (Figure 6I(b)) could be related to the unsteady state conditions and heterogeneous distribution of ATL-contaminated DW inflow at the beginning of filtration time. The same phenomenon was still observed in some graphs (Figure 6II(b),III(b)). It is recognisable that mainly the increase in PG dosage affected the treatment efficiency positively. Most of ECs were removed with the highest treatment efficiencies at the largest dose (500 mg of PG) except for DCF (Figure 6IV(b)) in which all doses showed similar effluent concentration profiles. One of the reasons for these unusual results of DCF decontamination could be attributed to the relatively low adsorption capacity of PG for DCF (around 82 mg-DCF/g-PG [19]). Besides, the PG-free sand column showed an outperformance over PG-sand filters (Figure 4d); therefore, the increase in PG loading was not significant in improving the targeted treatment. The order of treatment performance from the highest to lowest was as follows: 500 mg-PG/sand filter > 350 mg-PG/sand filter > 250 mg-PG/sand filter > 100-mg PG/sand filter.

In summary, by increasing the dosage of PG packed and mixed with sand, the effluent concentrations were decreased significantly, and this appeared more evidently in the larger cross-sectional-area columns. 

#### 3.2.4. Different Reactive Bed Heights at Fixed PG Dosages

**Figure 7:**
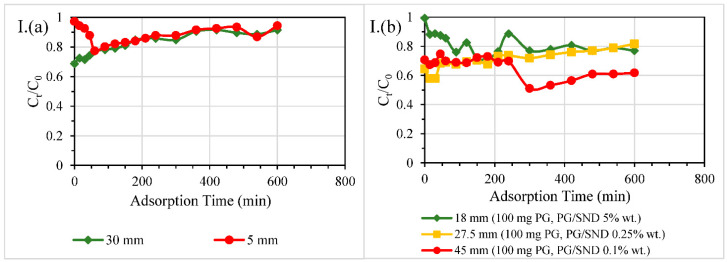

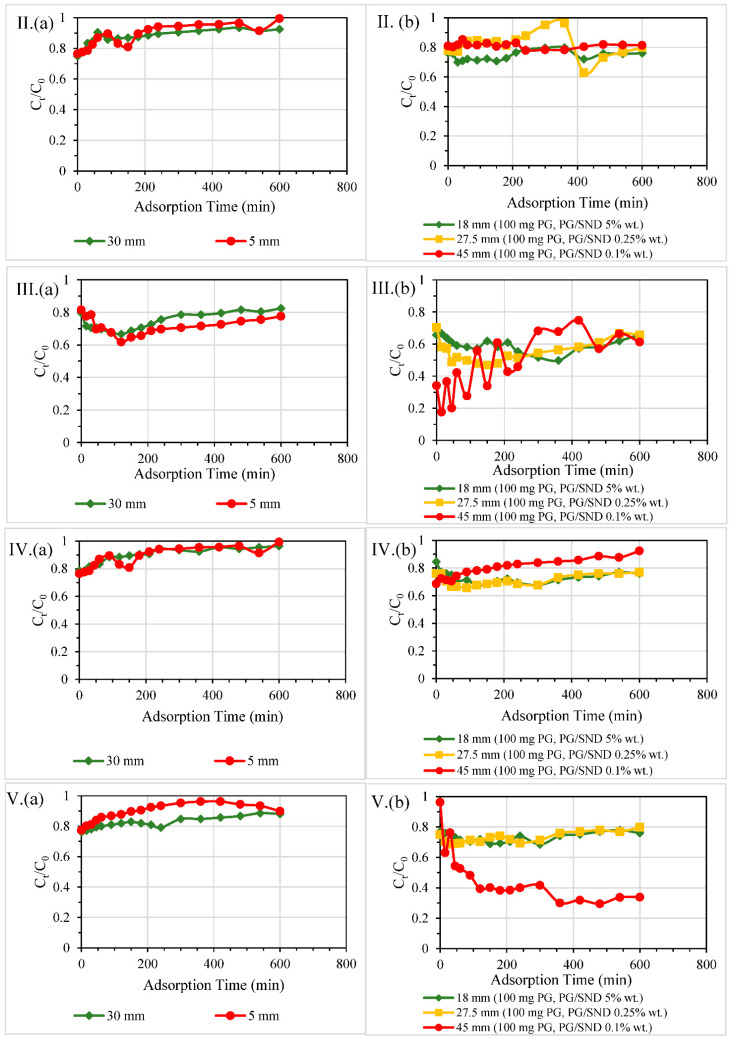

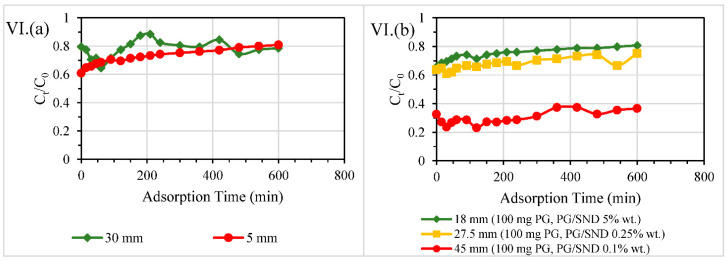
(**I**) ATL, (**II**) CBZ, (**III**) CIP, (**IV**) DCF, (**V**) GEM, (**VI**) IBP adsorption onto PG/SND filter medium of different heights in various column sizes (**a**) 18 mm ID (**b**) 40 mm ID. Experimental conditions: ECs-contaminated DW influent adjusted at 10.5 mg/L of EC; room temperature at 22 ± 3 °C.

Investigations on adsorption performance of PG filter medium at different packing heights of fixed PG dosage were carried out, using 50 mg PG in 18-mm ID column with two packing heights, 5 mm or 30 mm (PG/SND, 5% wt.) and using 100 mg PG in 40-mm ID column with three packing heights, 18 mm (PG/SND, 5% wt.), 27.5 mm (PG/SND, 0.25%) or 45 mm (PG/SND, 0.1%). The aforesaid filters were used to treat DW spiked with a certain EC at 10.5 mg/L and the treated effluent concentrations compared to the influent concentration of that EC were determined against the treatment time as shown in Figure 7.

The results given in Figure 7I–VI showed a significant decrease in effluent concentrations by increasing the height of PG packed and mixed with sand, and this was illustrated more clearly in the larger cross-sectional-area columns. In Figure 7I–VI(a), the increase in effective bed height has no substantial impact on the filtration performance of 18-mm filters and the change from 5 mm to 30 mm (with the same PG loading) did not considerably change the breakthrough curve. In larger size column tests, the effect of reactive bed height was demonstrated clearly in the 45-mm reactive bed height column (Figure 7I(b),III(b),V(b),VI(b)). Breakthrough curves of DCF-contaminated DW treatment using 18 mm, 27.5 mm, and 45 mm reactive bed columns nearly coincided with one another. The same behaviour occurred for CBZ-contaminated DW treatment, showing that the increase of reactive bed height did not assure an enhancement in filtration. However, in general, the considerable increase in the height of reactive bed packing resulted in a longer hydraulic retention time (HRT), which often led to an observed outperformance in filtration in comparison with the others. 

#### 3.2.5. Overall Efficiencies of Column Filters

Table 1, Table 2, Table 3, Table 4, Table 5 and Table 6 summarise the data presented above, including different cross-sectional areas, PG doses, and packing heights at a given dosage. Numerical values of HRT through the reactive layer and overall removal efficiency (ORE) calculated from the integration of differential EC component material balance are shown for better illustration and comparisons than the graphs. The ORE as an indication of the adsorption column (filter) performance is defined as the percentage of (mass) amount of EC removed/adsorbed through filtration during the whole operation time per total inlet of EC mass to the column filter. In general, the removal efficiency (RE) of a filter at a certain time (*t*) can be expressed by the following equation:

$$RE\%\;=\frac{C_{0}V\left(t\right)-\;\int_{C_{0}}^{C_{t}}C_{t}dV\left(t\right)}{C_{0}V\left(t\right)}\;\times \;100,$$

where the *RE* was evaluated by subtracting the EC effluent mass (the integral part) from the total EC influent mass $C_{0}V\left(t\right)$ and divided by that total EC influent mass. $C_{0}$ is the EC concentration $C_{t}$ at *t* = 0 and *V*(*t*) is the volume of water treated at a certain time *t*. For ORE calculation, the time (*t*) is taken as the total operation time of filters.

The same conclusions in Section 3.2 are drawn from Table 1, Table 2, Table 3, Table 4, Table 5 and Table 6 by comparing the numbers (HRT and ORE values). These tables demonstrate that the ORE of any EC increased by increasing the cross-sectional area at the same dosage (from 64.32 to 82.8% for ATL removal in Table 1; 71.5 to 85.2% for CBZ removal in Table 2; 76.7 to 85.85% for CIP removal in Table 3; 74.14 to 87.04% for DCF in Table 4; 73.6 to 89.73% for IBP in Table 5 and 73.75 to 91.13% for GEM in Table 6). For different doses, filters of large diameters (ID 40 mm), with the highest dose (500 mg PG (PG/SND 0.5% wt.)), and longest packing reactive bed height (45 mm) showed the longest HRT (ca. 47.5 min) and subsequently exhibited the highest ORE (90.07%, 90.27%, 91.42%, 73.57%, 92.02% and 86.38% for ATL, CBZ, CIP, DCF, IBP, and GEM). Even using different heights at the same PG dosage can enhance the ORE by increasing the reactive bed length as shown in Table 1, Table 2, Table 3, Table 4, Table 5 and Table 6. For instance, GEM removal in 18-mm filters was improved from 67.44% to 69.11% by increasing the packing length from 10 to 30 mm and enhanced gradually from 70.75% to 70.89% and then to 81.94% by increasing the reactive bed height from 18 to 27.5 and then to 45 mm.

Overall, the results in Table 1, Table 2, Table 3, Table 4, Table 5 and Table 6 showed the optimum conditions among the investigated parameters (column size, bed depth, material loading/dosage) for PG application as porous reactive filter media for ECs removal. For a stream containing a relatively high conc. of EC at 10.5 mg/L, the best result obtained using a PG mixed with sand layer filter with higher cross-sectional area, dose, packing height and correspondingly HRT (47.5 min) can reach 90% as an ORE and above.

#### 3.2.6. Real Samples and Interference Studies

**Figure 8:**
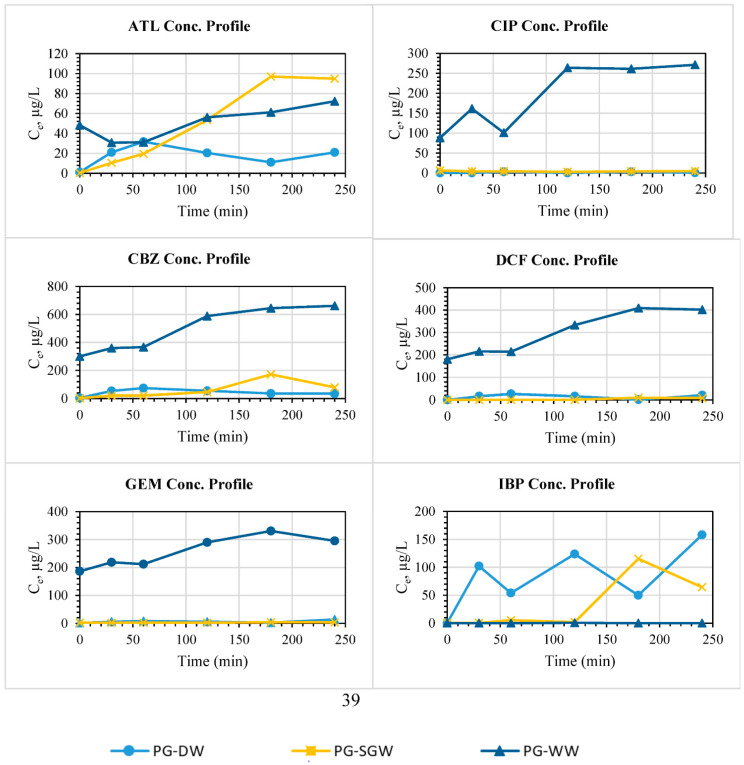
The breakthrough curve of EC adsorption onto PG in DW, SGW, and WW as backgrounds (pH 7.5; initial individual EC concentration: 1 mg/L; PG loading dosage: 100 mg).

The results of column studies on the filtration of DW, SGW, and WW bodies spiked with multiple ECs, each at 1 mg/L, using PG are shown in Figure 8. The presence of competing ions and organic matters in contaminated water have mixed effects on adsorption in real sample investigations based on the nature of interfering matter, adsorbent and water body type, and adsorption mechanism [42]. From our column studies, it was found that there was no negative interference with several ECs removal from two water bodies, DW and SGW. PG treated most of the ECs in the same manner, and the breakthrough curves shown in Figure 8 for both PG-DW and PG-SGW for the removal of CIP, CBZ, DCF, and GEM coincide with one another. It was observed that the effluent concentrations of ECs for contaminated WW filtration were higher than those of other water bodies. This may indicate the effect of competing ions such as ammonium and chlorides (Table S3) in reducing the adsorption capacity of PG for these contaminants. While this negative interference occurred in the column studies and was not found in batch tests [19], this could be attributed to the bactericidal properties of GBMs/sand [43], which were well distributed in the sand columns. In addition, the sand support could play a role in inhibiting the biodegradation of the ECs as it supported the GBMs and enhanced the availability of their contact surface area with the ECs, making their active sites available for water remediation. CIP, CBZ, DCF, and GEM were readily removed by filtration using PG (100 mg PG as a reactive bed of PG/SND mixture) having removal efficiencies above 99% against 6 mg/L of total ECs mixture and numerous common ions and organic matters in the case of SGW. 

## 4. Conclusions

This study assessed the efficacy of porous graphene material (PG) as a filter medium to remove six emerging contaminants (ECs), namely, atenolol (ATL), ciprofloxacin (CIP), carbamazepine (CBZ), Diclofenac (DCF), Gemfibrozil (GEM) and Ibuprofen (IBP), from different water bodies. All ECs were examined in column studies with different configurations (sand column, one-layer PG reactive bed column, and two-layer reactive bed column). The PG-sand column containing double layers of PG dose was proved to be the best and highly efficient. This improved performance could be related to the extended length of packing material, which facilitated more time for adsorption. For a column (40 mm ID × 100 mm H) containing a sand and double PG layers (500 mg total of PG), ECs removal efficiency was maintained above 90% for about 100 min. The sand assisted the adsorption process of ECs, especially DCF for which the adsorption capacity of sand reached 33.776 mg/g. In the further experiments, the performance of PG as filter media for ECs removal from water was assessed in the column studies under different conditions (such as size of column, packing heights, and adsorbent dosage). It was concluded that the adsorption performance of PG packing was improved in a larger cross-sectional-area column. At least 10% increase in the overall removal efficiency (ORE) was achieved via increasing the filter size from ID 18 mm to 40 mm. By increasing the height and dosage of PG packed and mixed with the sand, the effluent concentrations decreased significantly, and this appeared more evidently in the larger cross-sectional-area columns. By combining the effects of previous factors, the ORE of most of ECs by PG filters increased to around 90%. Finally, the filtration of ECs mixture at trace concentrations in various water matrices revealed no considerable interference with greywater constituents, and the treatment results of some ECs (CIP, CBZ, DCF, and GEM) removal were similar to those with distilled water body with removal efficiencies above 99%. On the contrary, the presence of competing ions and organic matter comparatively degraded PG filter performance to treat ECs in wastewater. Overall, an adsorption column filter with PG packing of optimised design and operation parameters could potentially be used as a highly efficient tertiary treatment unit for the removal of pharmaceutical contaminants. 

## Figures and Tables

**Table 1:** Hydraulic retention time and overall removal efficiency of ATL removal by column filters of different cross sectional areas, PG doses, and packing heights at constant dosage. Experimental conditions: ECs-contaminated DW influent adjusted at 10.5 mg/L of EC; room temperature at 22 ± 3 °C.

Contaminant	Test	Diameter of Column, mm	Material Dosage	Packing Height, mm	HRT through Reactive Layer, min	Overall Removal Efficiency %
**ATL**	**Different cross sectional areas**	18	500 mg PG	25	11.45	64.32
		40	500 mg PG	5	11.31	82.80
	**Different PG doses**	18	500 mg PG	25	11.45	64.32
		18	50 mg PG	10	4.58	67.455
		40	500 mg PG (PG/SND 0.5% wt.)	45	47.50	90.08
		40	100 mg PG (PG/SND 0.5% wt.)	18	19.00	67.58
		40	250 mg PG (PG/SND 0.5% wt.)	27.5	29.03	74.85
		40	350 mg PG (PG/SND 0.5% wt.)	30	31.67	73.34
	**Different packing heights at constant dosage**	18	50 mg PG (PG/SND 0.25% wt.)	30	6.41	69.00
		18	50 mg PG	10	4.58	67.46
		40	100 mg PG (PG/SND 0.5% wt.)	18	19.00	67.58
		40	100 mg PG (PG/SND 0.25% wt.)	27.5	29.03	71.85
		40	100 mg PG (PG/SND 0.1% wt.)	45	47.50	73.71

**Table 2:** Hydraulic retention time and overall removal efficiency of CBZ removal by column filters of different cross sectional areas, PG doses, and packing heights at constant dosage. Experimental conditions: ECs-contaminated DW influent adjusted at 10.5 mg/L of EC; room temperature at 22 ± 3 °C.

Contaminant	Test	Diameter of Column, mm	Material Dosage	Packing Height, mm	HRT through Reactive Layer, min	Overall Removal Efficiency %
**CBZ**	**Different cross sectional area**	18	500 mg PG	25	11.45	71.50
		40	500 mg PG	5	11.31	85.20
	**Different PG doses**	18	500 mg PG	25	11.45	71.50
		18	50 mg PG	10	4.58	64.92
		40	500 mg PG (PG/SND 0.5% wt.)	45	47.50	90.27
		40	100 mg PG (PG/SND 0.5% wt.)	18	19.00	70.27
		40	250 mg PG (PG/SND 0.5% wt.)	27.5	29.03	69.53
		40	350 mg PG (PG/SND 0.5% wt.)	30	31.67	75.42
	**Different packing heights at constant dosage**	18	50 mg PG (PG/SND 0.25% wt.)	30	6.41	67.03
		18	50 mg PG	10	4.58	64.92
		40	100 mg PG (PG/SND 0.5% wt.)	18	19.00	70.27
		40	100 mg PG (PG/SND 0.25% wt.)	27.5	29.03	67.25
		40	100 mg PG (PG/SND 0.1% wt.)	45	47.50	67.56

**Table 3:** Hydraulic retention time and overall removal efficiency of CIP removal by column filters of different cross sectional areas, PG doses, and packing heights at constant dosage. Experimental conditions: ECs-contaminated DW influent adjusted at 10.5 mg/L of EC; room temperature at 22 ± 3 °C.

Contaminant	Test	Diameter of Column, mm	Material Dosage	Packing Height, mm	HRT through Reactive Layer, min	Overall Removal Efficiency %
**CIP**	**Different cross sectional area**	18	500 mg PG	25	11.45	76.71
		40	500 mg PG	5	11.31	85.85
	**Different PG doses**	18	500 mg PG	25	11.45	76.71
		18	50 mg PG	10	4.58	70.42
		40	500 mg PG (PG/SND 0.5% wt.)	45	47.50	91.42
		40	100 mg PG (PG/SND 0.5% wt.)	18	19.00	76.32
		40	250 mg PG (PG/SND 0.5% wt.)	27.5	29.03	81.10
		40	350 mg PG (PG/SND 0.5% wt.)	30	31.67	86.93
	**Different packing heights at constant dosage**	18	50 mg PG (PG/SND 0.25% wt.)	30	6.41	72.18
		18	50 mg PG	10	4.58	70.42
		40	100 mg PG (PG/SND 0.5% wt.)	18	19.00	76.32
		40	100 mg PG (PG/SND 0.25% wt.)	27.5	29.03	78.08
		40	100 mg PG (PG/SND 0.1% wt.)	45	47.50	80.86

**Table 4:** Hydraulic retention time and overall removal efficiency of DCF removal by column filters of different cross sectional areas, PG doses, and packing heights at constant dosage. Experimental conditions: ECs-contaminated DW influent adjusted at 10.5 mg/L of EC; room temperature at 22 ± 3 °C.

Contaminant	Test	Diameter of Column, mm	Material Dosage	Packing Height, mm	HRT through Reactive Layer, min	Overall Removal Efficiency %
**IBP**	**Different cross sectional area**	18	500 mg PG	25	11.45	73.58
		40	500 mg PG	5	11.31	89.73
	**Different PG doses**	18	500 mg PG	25	11.45	73.56
		18	50 mg PG	10	4.58	70.08
		40	500 mg PG (PG/SND 0.5% wt.)	45	47.50	92.02
		40	100 mg PG (PG/SND 0.5% wt.)	18	19.00	70.13
		40	250 mg PG (PG/SND 0.5% wt.)	27.5	29.03	77.72
		40	350 mg PG (PG/SND 0.5% wt.)	30	31.67	82.30
	**Different packing heights at constant dosage**	18	50 mg PG (PG/SND 0.25% wt.)	30	6.41	70.56
		18	50 mg PG	10	4.58	70.08
		40	100 mg PG (PG/SND 0.5% wt.)	18	19.00	70.13
		40	100 mg PG (PG/SND 0.25% wt.)	27.5	29.03	72.94
		40	100 mg PG (PG/SND 0.1% wt.)	45	47.50	88.05

**Table 5:** Hydraulic retention time and overall removal efficiency of IBP removal by column filters of different cross sectional areas, PG doses, and packing heights at constant dosage. Experimental conditions: ECs-contaminated DW influent adjusted at 10.5 mg/L of EC; room temperature at 22 ± 3 °C.

Contaminant	Test	Diameter of Column, mm	Material Dosage	Packing Height, mm	HRT through Reactive Layer, min	Overall Removal Efficiency %
**DCF**	**Different cross sectional area**	18	500 mg PG	25	11.45	74.14
		40	500 mg PG	5	11.31	87.04
	**Different PG doses**	18	500 mg PG	25	11.45	74.14
		18	50 mg PG	50	4.58	64.33
		40	500 mg PG (PG/SND 0.5% wt.)	45	47.50	73.57
		40	100 mg PG (PG/SND 0.5% wt.)	18	19.00	70.96
		40	250 mg PG (PG/SND 0.5% wt.)	27.5	29.03	72.38
		40	350 mg PG (PG/SND 0.5% wt.)	30	31.67	72.46
	**Different packing heights at constant dosage**	18	50 mg PG (PG/SND 0.25% wt.)	30	6.41	66.40
		18	50 mg PG	50	4.58	64.33
		40	100 mg PG (PG/SND 0.5% wt.)	18	19.00	70.96
		40	100 mg PG (PG/SND 0.25% wt.)	27.5	29.03	71.64
		40	100 mg PG (PG/SND 0.1% wt.)	45	47.50	68.01

**Table 6:** Hydraulic retention time and overall removal efficiency of GEM removal by column filters of different cross sectional areas, PG doses, and packing heights at constant dosage. Experimental conditions: ECs-contaminated DW influent adjusted at 10.5 mg/L of EC; room temperature at 22 ± 3 °C.

Contaminant	Test	Diameter of Column, mm	Material Dosage	Packing Height, mm	HRT through Reactive Layer, min	Overall Removal Efficiency %
**GEM**	**Different cross sectional area**	18	500 mg PG	25	11.45	73.75
		40	500 mg PG	5	11.31	91.14
	**Different PG doses**	18	500 mg PG	25	11.45	73.75
		18	50 mg PG	10	4.58	67.44
		40	500 mg PG (PG/SND 0.5% wt.)	45	47.50	86.38
		40	100 mg PG (PG/SND 0.5% wt.)	18	19.00	70.75
		40	250 mg PG (PG/SND 0.5% wt.)	27.5	29.03	70.74
		40	350 mg PG (PG/SND 0.5% wt.)	30	31.67	74.62
	**Different packing heights at constant dosage**	18	50 mg PG (PG/SND 0.25% wt.)	30	6.41	69.11
		18	50 mg PG	10	4.58	67.44
		40	100 mg PG (PG/SND 0.5% wt.)	18	19.00	70.75
		40	100 mg PG (PG/SND 0.25% wt.)	27.5	29.03	70.88
		40	100 mg PG (PG/SND 0.1% wt.)	45	47.50	81.94

## Data Availability

The research data supporting this publication are given within this paper and as supplementary material.

## Supplementary Materials

The following are available online at https://www.mdpi.com/2079-4991/11/1/79/s1, Table S1: Chemicals used for synthetic grey water preparation, Table S2: Water quality and characteristics of the synthesised greywater, Table S3: Water quality of the effluent from secondary settlement tank prior to tertiary biological aerated flooded filter (BAFF) treatment unit, existing at Countess Wear Wastewater Treatment Works, South West Water Co., Exeter, Devon, UK, Table S4: Target compounds and their structures, main m/z ions, fragmentor and collision energy voltages, and average retention time for LC-MS analysis, Figure S1: (a) A photo of actual column experiment apparatus; (b) packing arrangement of columns for single (left) and double layers (right) of adsorbent, Figure S2: ATL, CIP, and DCF adsorption onto sand at different dosages after 24 h.
