# Supplementary material for: Performance Evaluation of Porous Graphene as Filter Media for the Removal of Pharmaceutical/Emerging Contaminants from Water and Wastewater
Source: Nanomaterials (Basel). 2021 Jan 1;11(1):79. doi: 10.3390/nano11010079 (PMC7824533; doi:10.3390/nano11010079)
Supplement: Supplementary file 1 [file nanomaterials-11-00079-s001.pdf]

Supplementary Information Material

Performance Evaluation of Porous Graphene as Filter Media for the Removal of Pharmaceutical/Emerging Contaminants from Water and Wastewater

Ahmed M.E. Khalil [1,2,*], Fayyaz A. Memon [1,*], Tanveer A. Tabish [1,3], Ben Fenton [1], Deborah Salmon [4], Shaowei Zhang [1] and David Butler [1]

[1] College of Engineering, Mathematics and Physical Sciences, University of Exeter, Exeter EX4 4QF, UK; t.a.tabish2@exeter.ac.uk (T.A.T.); bf290@exeter.ac.uk (B.F.); S.Zhang@exeter.ac.uk (S.Z.); D.Butler@exeter.ac.uk (D.B.)

[2] Department of Chemical Engineering, Faculty of Engineering, Cairo University, Giza 12613, Egypt

[3] UCL Cancer Institute, University College London, Bloomsbury, London WC1E 6DD, UK

[4] College of Life and Environmental Sciences, University of Exeter, Exeter, Devon EX4 4QD, UK; D.L.Salmon@exeter.ac.uk

* Correspondence: a.a.khalil@exeter.ac.uk (A.M.E.K.); f.a.memon@exeter.ac.uk (F.A.M.); Tel.: +44-1392-724048

Table S1. Chemicals used for synthetic grey water preparation.

Product	Quantity Used / Concentration	Product	Quantity Used / Concentration
Calcium Chloride	36 mg/L	Magnesium Sulphate	126 mg/L
Kaolin	57.5 mg/L	Monopotassium Phosphate	54 mg/L
Cellulose	57.5 mg/L	Iron (III) Chloride	30 mg/L
Humic Acid	15 mg/L	Boric Acid	3.0 mg/L
Sodium Chloride	70 mg/L	Manganese (II) Chloride	3.0 mg/L
Sodium Hydrogen Carbonate	55 mg/L	Zinc Sulphate	10 mg/L
Distilled water	<1L	Copper Sulphate	4.5 mg/L
Potassium Nitrate	50 mg/L	Ammonium Molybdate tetrahydrate	1.0 mg/L
Calcium Nitrate	80 mg/L	Cadmium Oxide	7.0 mg/L
Secondary Treatment Effluent with microbial content	60 ml/L	Nickel Oxide	0.1 mg/L
Sodium Phosphate Monobasic	130 mg/L	Chromium (III) nitrate	38 mg/L
Lead (II) Oxide	0.9 mg/L	Sodium Sulphate	15 mg/L

Tables S2. Water quality and characteristics of the synthesised greywater.

Analysis	Value	Unit
BOD	23	mg/L
COD	70.3	mg/L
Ammonia as Nitrogen	1.08	mg/L
Nitrate as Nitrogen	5.3	mg/L
Ortho-phosphate-phosphorus	28.4	mg/L
pH	7.5	
Redox potential	-6.8	mV
Turbidity	103.6	NTU
Electronic conductivity	543.8	µS/cm
Dissolved oxygen	10	mg/L

Table S3. Water quality of the effluent from secondary settlement tank prior to tertiary biological aerated flooded filter (BAFF) treatment unit, existing at Countess Wear Wastewater Treatment Works, South West Water Co., Exeter, Devon, UK.

Analysis	Value	Unit
pH	7.1	pH units
BOD	4	mg/L
Solids Suspended at 105 ^{o}C by Gravimetry	13	mg/L
Solids Suspended at 500 ^{o}C by Gravimetry	5	mg/L
Aluminium as Al by ICPOES[1]	<0.2	mg/L
Iron as Fe by ICPOES	<0.45	mg/L
Manganese as Mn by ICPOES	<0.08	mg/L
Copper as Cu by ICPOES	<0.004	mg/L
Zinc as Zn by ICPOES	0.0102	mg/L
Lead as Pb by ICPOES	0.0265	mg/L
Cadmium as Cd by ICPOES	<0.003	mg/L
Chromium as Cr by ICPOES	<0.024	mg/L
Nickel as Ni by ICPOES	<0.01	mg/L
Nitrogen Total Oxidised as N by Colorimetry	2.0	mg/L
Ammonia as N by Colorimetry	13	mg/L
Chloride as Cl- by Colorimetry	48	mg/L
Phosphorus Tot as P by ICPOES	0.530	mg/L

[1] ICPOES - Inductively coupled plasma - optical emission spectrometer

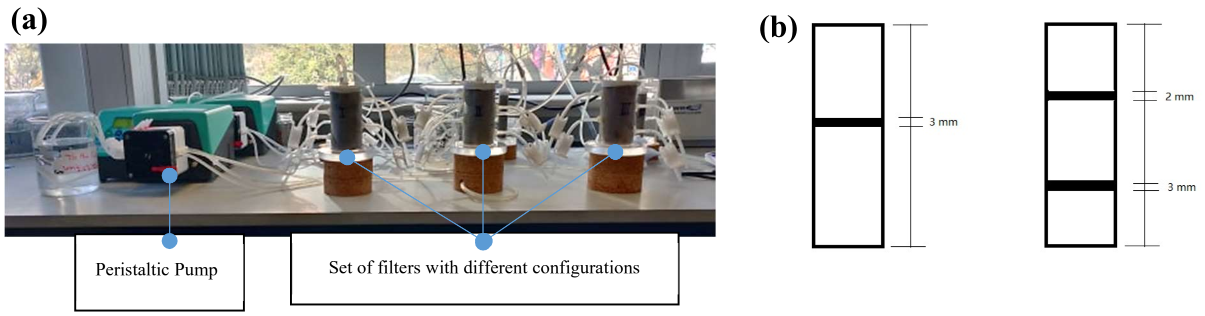

Figure S1. (a) A photo of actual column experiment apparatus; (b) packing arrangement of columns for single (left) and double layers (right) of adsorbent.

Table S4. Target compounds and their structures, main m/z ions, fragmentor and collision energy voltages, and average retention time for LC-MS analysis.

Drug	Chemical Structure	Precursor m/z	ID Product m/z	Qualifier Product m/z	Fragmentor Voltage (kV)	Collision Energy Voltages (kV)	Average RT (min)
**Atenolol**	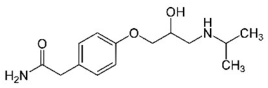	267.2	145.1	190.1	100	29 & 17	1.71
**Carbamazepine**	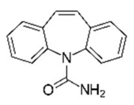	237.1	194.1	179.1	140	21 & 35	2.58
**Ciprofloxacin**	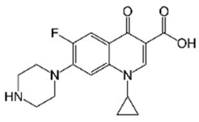	332.1	288.2	314.1	100	17 & 21	5.28
**Diclofenac**	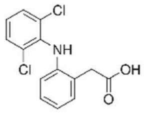	296.0	214.1	250.0	80	35 & 9	7.03
**Gemfibrozil**	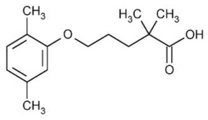	251.2	129.1	55.1	60	9 & 33	7.41
**Ibuprofen**	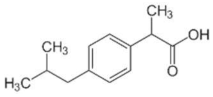	207.1	161.0	119.1	60	9 & 21	6.63

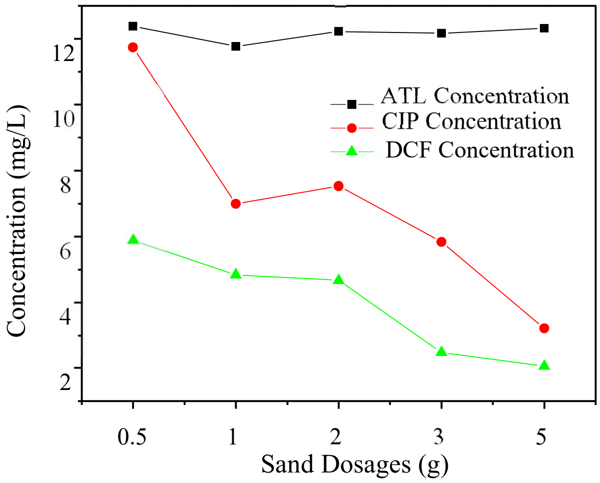

Figure S2. ATL, CIP, and DCF adsorption onto sand at different dosages after 24 hrs.
